# Simplifying the Treatment of Bone Atrophy in the Posterior Regions: Combination of Zygomatic and Wide-Short Implants—A Case Report with 2 Years of Follow-Up

**DOI:** 10.1155/2016/5328598

**Published:** 2016-10-27

**Authors:** Fernanda Faot, Geninho Thomé, Amália Machado Bielemann, Caio Hermann, Ana Cláudia Moreira Melo, Luis Eduardo Marques Padovan, Ivete Aparecida de Mattias Sartori

**Affiliations:** ^1^School of Dentistry, Federal University of Pelotas (UFPEL), Pelotas, RS, Brazil; ^2^Implantology Team, Latin American Institute of Dental Research and Education (ILAPEO), Curitiba, PR, Brazil; ^3^Graduate Program in Dentistry, School of Dentistry, Federal University of Pelotas, Pelotas, RS, Brazil

## Abstract

The rehabilitation of maxillary and mandibular bone atrophy represents one of the main challenges of modern oral implantology because it requires a variety of procedures, which not only differ technically, but also differ in their results. In the face of limitations such as deficiencies in the height and thickness of the alveolar structure, prosthetic rehabilitation has sought to avoid large bone reconstruction through bone grafting; this clinical behavior has become a treatment system based on evidence from clinical scientific research. In the treatment of atrophic maxilla, the use of zygomatic implants has been safely applied as a result of extreme technical rigor and mastery of this surgical skill. For cases of posterior mandibular atrophy, short implants with a large diameter and a combination of short and long implants have been recommended to improve biomechanical resistance. These surgical alternatives have demonstrated a success rate similar to that of oral rehabilitation with the placing of conventional implants, allowing the adoption of immediate loading protocol, a decrease in morbidity, simplification and speed of the treatment, and cost reduction. This case report presents complete oral rehabilitation in a patient with bilateral bone atrophy in the posterior regions of the maxilla and mandible with the goal of developing and increasing posterior occlusal stability during immediate loading.

## 1. Introduction

The osseointegrated implants to support fixed prostheses revolutionized the rehabilitation treatment of totally and partially edentulous patients. However, in clinical situations where there is limited bone availability, the surgeon must often resort to bone grafting procedures, which prolong treatment time and increase cost and morbidity [1–3].

Bone graft reconstruction techniques inevitably present a component of risk because they require a precise surgical technique, a good quality of soft tissues that overlie the graft, patient cooperation, and general good health that favors recovery [4]. As these conditions are not always present in a single patient, complications such as graft contamination or exposure can lead to partial or total loss of the graft, resulting in an unsuccessful treatment that may include deleterious effects [5]. Even in cases where the treatment evolves without major complications and the possibility of installing a fixed prosthesis is given a favorable prognosis, doubts still remain in relation to both the stability of the results and the maintenance of the bone structure and soft tissues [6].

With regard to these problems, clinical strategies have been proposed to increase the success rate of implants installed in critical sites of bone atrophy that include the use of short implants with a wide diameter [7, 8], implants with a rough surface that increases the contact between the bone and the implant [9, 10], an increase in the number of implants [11–13], and even a combination of short and long implants to improve the biomechanical resistance to tension and occlusal forces [14–16].

Especially for posterior maxilla atrophy rehabilitation, the development and use of zygomatic implant [17–21] in conjunction with conventional accessory implants on the anterior region has proven a viable alternative [22–24] because it simplifies treatment by using less invasive surgeries and reduces the cost and time of treatment. In addition, this treatment has demonstrated a favorable prognosis and a success rate similar to that of conventional implants [25, 26].

Regarding this scenario, the purpose of this clinical case report is to present and discuss the biomechanical aspects related to oral rehabilitation in a patient with bilateral bone atrophy in the posterior regions of the maxilla and mandible with the goal of developing and increasing posterior occlusal stability during immediate loading.

## 2. Case Report

**Figure 1:**
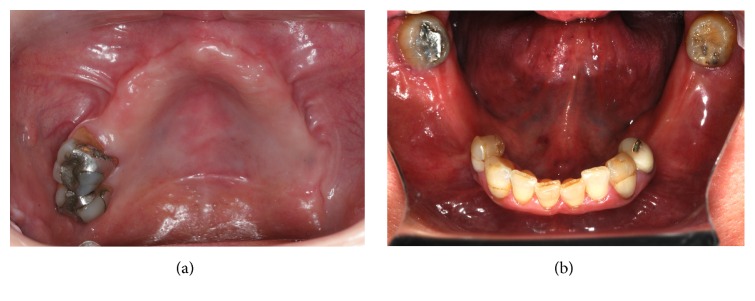
Clinical view at the initial appointment. Occlusal view of maxillary (a) and mandibular arch (b).

**Figure 2:**
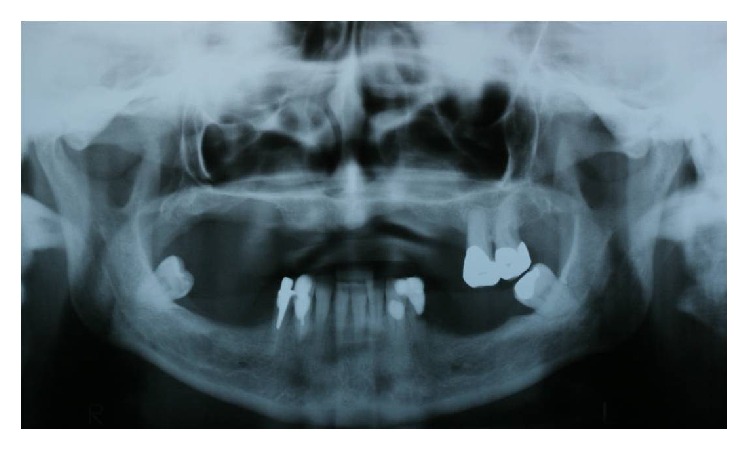
Panoramic radiograph from the initial examination.

**Figure 3:**
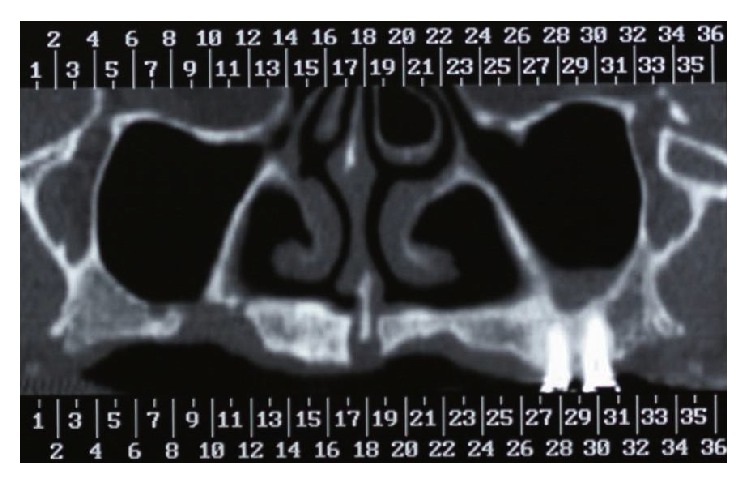
Computed tomography of maxilla. Distance between reconstructions: 3 mm.

**Figure 4:**
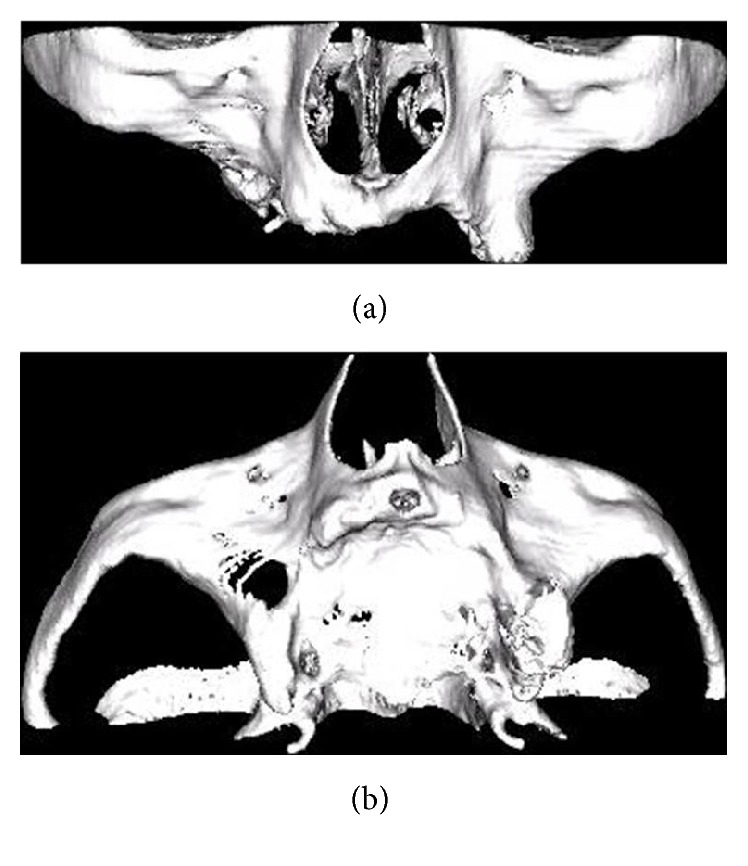
Maxilla 3D reconstruction in frontal (a) and occlusal view (b).

Patient I.M.S. (female, 50 years old) checked into ILAPEO* (Latin American Institute of Research and Education in Dentistry)* to undergo oral rehabilitation treatment. The patient presented with a good state of general health with partial edentulism of the upper and lower jaw (Figure 1(a)) and with removable partial prostheses. In the upper jaw, she had a provisional partial prosthesis and, in the lower jaw, a class III removable partial denture was seated on the third molars in an unfavorable position by distal retainers. The patient's main complaint was the lack of stability, retention of the upper removable partial denture, the positioning of the lower third molars, the sensitivity of element 34 due to little bone support, and the difficulty of using the inferior prosthesis, which frequently injured the adjacent soft tissues. After clinical and radiographic analysis by panoramic radiography (Figure 2), poor bone availability in the maxilla and posterior mandible was observed and additionally a computed tomography was requested to plan the case in greater detail (Figure 3). Due to the extreme maxillary atrophy in the right side (including a radiographic image suggesting oral-antral communication), the indication for reconstructive procedures did not have a favorable prognosis as it can be also observed in the 3D reconstruction image (Figure 4). For this case, an anchoring technique combining conventional and zygomatic implants could be an alternative solution for rehabilitation; extraction of elements 25 and 26 was suggested and was subsequently accepted by the patient. In the lower arch, the extraction of elements 38, 34, and 48 was also indicated together with a combination of screw retained fixed partial dentures (FPDs).

Prior to the installation of the implants, a prosthetic preparation was performed and included the recording and assembly of the upper teeth performed on a trial basis without anterior vestibular coverage to diagnose the lip support that the FPD would provide. With the patient's approval, this diagnostic assemblage was duplicated, and a multifunctional guide was obtained.

In the atrophic maxilla, to install the zygomatic implants, an intravenous general anesthesia was induced along with preparation for surgery using a local anesthetic based on 2% lidocaine hydrochloride with adrenaline at 1 : 100,000. Two zygomatic implants (Neodent Implante Osseointegrável, Curitiba, PR, Brazil) of 45 mm were installed with rotation around 800 rpm and their respective prosthetic abutments of 3.0 mm were installed and tightened using a mechanical torque limiter with 20 N/cm. In addition, to guarantee the Roy Polygon creation orientating the force distribution in the maxilla, four cylindrical implants (Titamax Cone Morse, Neodent Implante Osteointegrável, Curitiba, PR, Brazil) were also installed with diameter of 3.75 mm and a length of 9 mm for elements 11 and 21, 11 mm for element 22, and 13 mm for element 13. The clamping obtained a torque greater than 45 N/cm, showing primary stability that was sufficient for the use of immediate load in the maxilla. Besides, the impression was performed using the multifunctional guide technique, which consists of joining the guide to the impression posts that were previously splinted using self-curing acrylic resin (Pattern Resin, GC America, IL, USA). Afterwards, the interocclusal record was refined by using three points of self-curing acrylic resin after confirming the vertical occlusal dimensions provided by the multifunctional guide record and the material injected between the transferors by a molding syringe. After polymerization of the materials, the screws of the impression posts were loosened, and the multifunctional guide, which had functioned as a molding tray and an interocclusal record, was renewed and taken to the prosthetic laboratory to manufacture a full arch fixed implant-supported prosthesis. Afterwards, the prosthesis was installed with immediate load protocol.

Within the lower posterior edentulous spaces on both sides, cylindrical implants were installed (Titamax CM, Neodent Implante Osteointegrável, Curitiba, PR, Brazil) combined with shorter and wider implants (Titamax WS, Neodent Implante Osseointegrável, Curitiba, PR, Brazil) in the distal ends because of mandibular bone atrophy in these areas. In this case, they were installed with the goal of increasing posterior occlusal stability, avoiding the use of distal cantilevers, and favoring a more uniform distribution of occlusal charges during chewing. These short implants with wide diameter platforms for the cortical bone have the advantage that their cervical diameters correspond to the diameter of the implant's body, favoring the uniform distribution of occlusal charges during chewing. Moreover, the high cutting power of their angled tips follows the exact same path as that of the pilot drill tip, providing a perfectly fitted installation at the site of the implant and avoiding empty spaces. Specifically, these implants were maintained around 2 mm under the future gingival margin towards the cement enamel junction.

**Figure 5:**
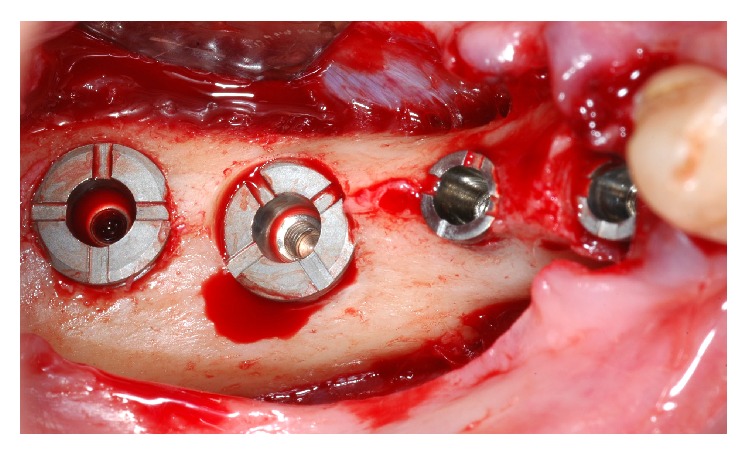
Wide-short implants installed at the bone level.

The surgical sequences for perforation to install the conventional implants followed the conventional protocol of progressive diameters with rotation around 1500 rpm and 300 rpm for short implants under abundant irrigation, paying attention to the mesiodistal and buccolingual position of the implant. The conventional cylindrical implants installed had a diameter of 3.75 mm and a width ranging from 7 to 17 mm: 7 mm for elements 36 and 45, 15 mm for element 44, and 17 mm for element 35. Due to a limitation of bone height in the posterior extremity, short implants were installed with a length of 5 mm and a diameter of 5 mm for the region corresponding to element 36 and of 6 mm for the 37 and 47 regions (Figure 5).

**Figure 6:**
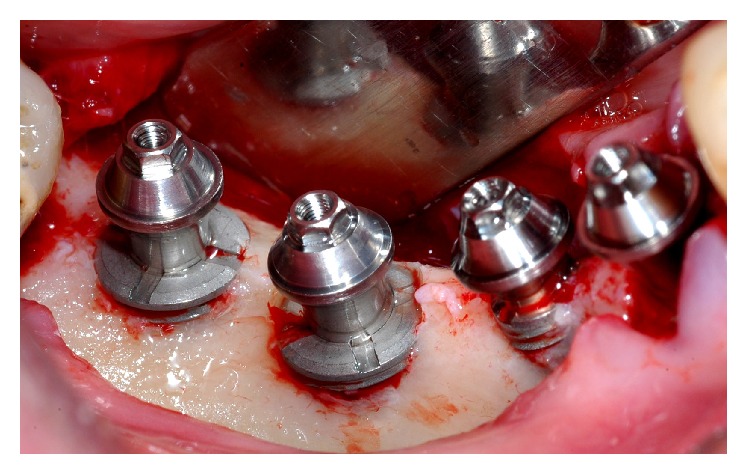
Abutments for multiple prosthesis installed during the surgery.

Primary stability was also obtained in the mandibular arch and the heights of the mini conical pillars were selected (WS CM, Neodent Implante Osseointegrável, Curitiba, PR, Brazil) and installed (Figure 6) using a torque of 32 N·cm. Afterwards, the impression of the lower arch was made using a perforating tray after installing the square impression posts for mini conical pillars that were splinted using self-curing acrylic resin.

**Figure 7:**
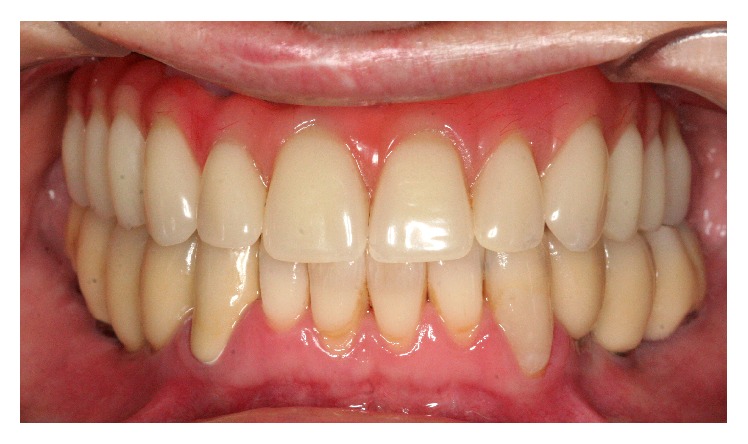
Frontal view of final restoration.

**Figure 8:**
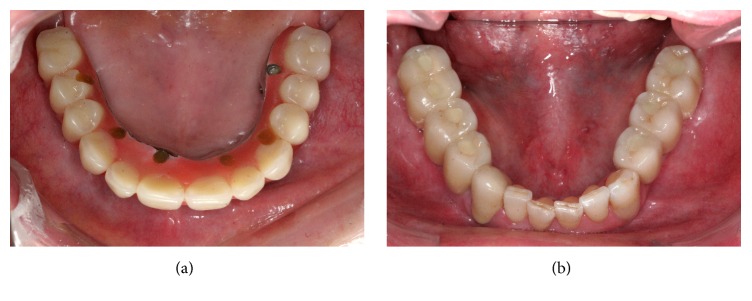
Occlusal view of final restoration: (a) maxilla and (b) mandible.

**Figure 9:**
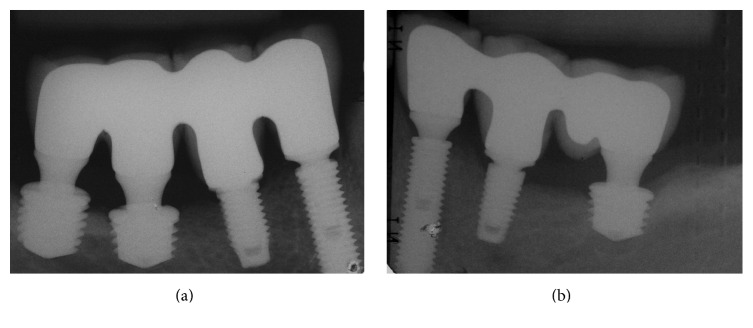
Periapical radiographs at the prosthesis installation session in the mandibular arch: (a) right and (b) left side.

**Figure 10:**
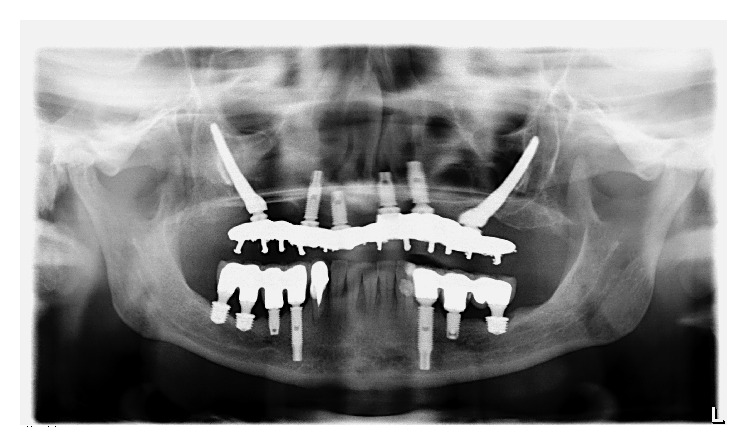
Panoramic radiograph at 2 years of follow-up.

After obtaining the impression (Speedex Light Body, Coltene, Vigodent SA Indústria e Comércio, RJ, Brazil) two provisional partial lower fixed dentures in acrylic resin were constructed. During the installation of the fixed dentures, periapical radiographs were performed in both sides and an occlusal adjustment was performed to establish simultaneous bilateral occlusal contacts in relation to the centric occlusion and the anterior guide. Procedures for the definitive lower prostheses were performed at the same time in both sides after three months and consisted of the following: obtaining a new impression, performing a radiographic test and evaluation of the metallic infrastructures, and registering the interocclusal record. Subsequently, a ceramic trial was performed and partial fixed denture prostheses (FDPs) were installed using a torque of 10 N·cm in the prosthetic screws (Figures 7 and 8). The occlusal adjustment also aimed to establish a mutually protected occlusion. The final periapical radiographic preservation (Figure 9) and 2 years of follow-up can be observed in the panoramic radiographic (Figure 10).

## 3. Discussion

The implants used in this clinical study have a morse taper connection. These implants have prosthetic abutments with a concave format design, associated with various biological advantages such as the preservation of the peri-implant bone and improved soft tissue quality [27]. The concave part of the prosthetic abutment allows the collagen fibers to fill the created space, resulting in a fabric necklace that will act as an effective attachment for connective tissue.

Prosthetic advantages ensure better stability of the prosthetic component and improvement in the biological aspect to reduce bone loss. The better mechanical stability and fixation of the prosthesis reduce rotational movement, resulting in higher resistance to screw loosening. It also reduces the clearance between the implant and the middle pillar and improves the junction and the implant abutment's bacterial seal [27]. However, this system also has some disadvantages. It demands greater accuracy in the preparation of the surgical bed and larger surgical care and there is less versatility with respect to prosthetic components for external hexagon connections [28].

Although the zygomatic implant technique is not considered a simple and common procedure in the clinical practice [17, 18, 20], it could be considered as an alternative to bone reconstructive procedures (grafts) and moreover as an excellent option of rehabilitation treatment for maxillary atrophy when combined with implants placed in the premaxilla [25, 26] to complete the biomechanical polygon. This biomechanical set will promote stability by allowing the vector cancellation of lateral forces considered deleterious to the zygomatic implants, since they are long and have a sharp lever arm due to the inclination of 45° between the platform and the body of anchorage [29, 30]. In addition, the choice from conventional, transepithelial, or tapered mini-pillar abutments is crucial, because of its position at the head of the implant, which will depend on the prosthetic connection and its respective prosthetic cylinders. Thus, it is preferable to use lower prosthetic abutments, thereby facilitating sculpturing of the metal structure and reducing the total volume of the final prosthesis.

The acceptance of the zygomatic implant technique by patients has increased because the need for grafts is eliminated, and there is a possibility of combining zygomatic implants with immediate loading [31]. In addition, factors such as the age of the patient, the time, the cost, and the morbidity may also guarantee predictability [32]. The failures indices reported in previous clinical studies are low, and most were detected at the abutment connection phase (6 months after the surgery of implant placement) or before [33]. It is also important to remember that the success rate is directly related to the experience and technical skills of the surgical team.

The patient's satisfaction with fixed prostheses supported by zygomatic implants in relation to comfort, stability, ability to talk, easiness to clean, aesthetics, and functionality has been similar to that related by patients rehabilitated using fixed prostheses with conventional implant [34, 35]. Another important issue is that, due to the anatomical limitations of the patient, this technique should be recommended to treat patients with maxillary bone atrophy who accept the rehabilitation required by the degree of atrophy because this procedure can result in metal-plastic prostheses with pink acrylic resin (flange exposition) in order to compensate horizontal and vertical discrepancies. As many patients expect to receive fixed prostheses with naturally sized teeth and with an emergence of gingival tissue, it is fundamental to the treatment's success that cases should start with prior prosthetic preparation. This would allow the surgeon to diagnose the degree of absorption and assess the relation of the interarches and would allow the patient to visualize these factors. The various therapeutic possibilities for resolving these cases should be weighed by the professional, emphasizing to the patient their advantages and limitations.

Implants of larger diameter are recommended in the posterior region of the mandible and in bones with lower quality or reduced volumes. The latter aims to increase the tolerance to occlusal force, preventing initial instability and promoting a more favorable tension balance around the bone [36]. Theoretically, wide diameter implants anchored in cortical bones can achieve an increase in stability proportional to its diameter [37] because of the anchorage in the lingual or the buccal cortical bone. The reduced height would then be partially compensated by an increase in the implant diameter, producing a larger superficial contact area between the bone and the titanium and resulting in a lower failure rate for short implants, mainly in the posterior atrophic mandible region [38].

The main downside of the larger diameter is a larger volume of bone substituted by titanium, which can induce bone loss around the implant. In addition, the posterior region of the mandible typically has dense cortical tissue with low vascularization and remodeling/formation. The latter suggests that the risk of initial stability loss can be reduced during the remodeling phase [39]. Finally, the available surface area for implants in most systems is limited, reducing its applicability and such systems have lower resistance to occlusal forces.

Concerning the treatment of atrophy of the posterior mandible with short implants, a high clinical success rate (ranging from 80 to 100%) has been reported in prospective, retrospective, and case report follow-up studies [40–45]. Furthermore, differences have not been observed between short implants and other modalities of prosthetic rehabilitation of severe resorptive mandibles [40–42]. Thus, these studies are providing reason for the reevaluation of the results of previous studies that indicate that short implants can properly support most of prosthetic restorations.

The longevity of short implants relies on prosthetic factors such as crown, implant ratio, occlusal table width, occlusion with normal maxillomandibular relationship towards buccolingual orientation, rigid union of the implants through metal structures, and antagonist dentition [12]. Occlusal and anatomic factors in relation to the quality and quantity of the remaining bone, the length of the mesiodistal edentulous space, and the maxillomandibular relationship should also be carefully evaluated [16]. The complications observed in this kind of treatment can be related to the increase of the crown height, a higher bite force in the posterior regions, and low bone density [12]. Furthermore, literature has shown that most of the cases recording a loss of these implants occur in the first year before the patient receives the prosthetic loading [40–42] and one factor that directly influences the osseointegration and survival rate of these types of implants is their rigid union through a metallic infrastructure when the prostheses are installed [9, 46].

Therefore, based on the scientific literature, we infer that the prognosis of the clinical case reported herein, referring to the rehabilitation of the posterior mandible region, can be considered favorable and well established because in the right free end it was combined with implants of 3.75 × 17 mm, 3.75 × 7 mm, and 6.0 × 5 mm, which resulted in a bone contact area of approximately 572.42 mm^2^ while in the left free end there were implants of 3.75 × 15 mm, 3.75 × 7 mm, 5.0 × 5 mm, and 6.0 × 5 mm, and the bone contact area was 509.32 mm^2^. Moreover, the rigid union through a metallic infrastructure with immediate function was considered a positive factor during the osseointegration period.

## 4. Conclusion

Zygomatic and short implants are a reality and make the rehabilitation of areas with severely low bone availability possible. These treatment options offer the possibility of reducing surgical procedures such as sinus lifting, bone grafts, transposition of the mandibular nerve, and positioning in areas of reduced prosthetic space and the possibility of avoiding cantilever in posterior regions.
